# The impact of tensioning device mal-positioning on strand tension during Anterior Cruciate Ligament reconstruction

**DOI:** 10.1186/1749-799X-6-33

**Published:** 2011-06-28

**Authors:** Rajesh Maharjan, John J Costi, Richard M Stanley, David Martin, Trevor C Hearn, John R Field

**Affiliations:** 1 Comparative Orthopaedic Research Surgical Facility, School of Medicine, Flinders University, Bedford Park, 5042, South Australia, Australia; 2 Flinders Medical Devices and Technologies - Biomechanics and Implants Group, School of Computer Science, Engineering and Mathematics, Flinders University, South Australia, Australia; 3 Sportsmed, Stepney, South Australia, Australia

## Abstract

**Background:**

In order to confer optimal strength and stiffness to the graft in Anterior Cruciate Ligament (ACL) reconstruction, the maintenance of equal strand tension prior to fixation, is desired; positioning of the tensioning device can significantly affect strand tension This study aimed to determine the effect of tensioning device mal-positioning on individual strand tension in simulated cadaveric ACL reconstructions.

**Methods:**

Twenty cadaveric specimens, comprising bovine tibia and tendon harvested from sheep, were used to simulate ACL reconstruction with a looped four-strand tendon graft. A proprietary tensioning device was used to tension the graft during tibial component fixation with graft tension recorded using load cells. The effects of the tensioning device at extreme angles, and in various locking states, was evaluated.

**Results:**

Strand tension varied significantly when the tensioning device was held at extreme angles (p < 0.001) or in 'locked' configurations of the tensioning device (p < 0.046). Tendon position also produced significant effects (p < 0.016) on the resultant strand tension.

**Conclusion:**

An even distribution of tension among individual graft strands is obtained by maintaining the tensioning device in an unlocked state, aligned with the longitudinal axis of the tibial tunnel. If the maintenance of equal strand tension during tibial fixation of grafts is important, close attention must be paid to positioning of the tensioning device in order to optimize the resultant graft tension and, by implication, the strength and stiffness of the graft and ultimately, surgical outcome.

## Background

Surgeons increasingly favour reconstruction of the anterior cruciate ligament with the multi-strand tendon autograft in preference to bone-patella tendon-bone grafts (BPTB) because of the relatively low complication rate [1] and availability of improved fixation methods; equally tensioned quadrupled hamstring tendon (QHT) grafts have been shown stronger and stiffer than BPTB grafts [2-4]; Initial graft tension plays a vital role in maintaining joint kinematics and in situ forces in the graft during knee motion [5,6]. The application of excessive intra-operative tension can precipitate joint stiffness, the development of abnormal stresses on the articular cartilage and menisci, and which may also interfere with graft revascularization [7-9]. Conversely, inadequate graft tension will lead to excessive joint laxity [3]. To maintain optimum biomechanical properties it appears important to generate, and maintain, similar tension in all four strands of the QHT graft at the time of graft tensioning and tibial fixation [10-12].

Currently, there is no consensus regarding the amount of tension to apply to a graft when it is secured [1]. An initial tension of 44N is considered optimum by some, but there is no empirical evidence for this argument [13,14]. Restoration of anterior translation to within 3 mm of the native ACL condition, after cyclic loading, required approximately 68 N initial tension to be applied [15]. Graft tensioning has been evaluated in numerous cadaveric studies [7,15-19] , with considerable variation in graft tension observed between surgeons, prompting the suggestion that graft tension should be more accurately measured and controlled intra-operatively [17]; Gertel et al [20] demonstrated that the direction of tensioning and the flexion angle of the knee at which the tension was applied also plays a significant role in the initial graft tension.

Various techniques have been used to maintain uniform tension in all strands of a QHT graft. Bellemans et al [21] demonstrated the use of one spiked staple to fix the hamstring tendon to the tibia in order to maintain the appropriate tension prior to introduction of an interference screw. Hamner et al [10] produced equal tension in the strands by applying weights. Commercially available tensioning devices can reportedly produce and maintain equal tension in the strands of QHT. In principle, when the tensioning device is pulled it exerts equal tension in all of the strands. However, when the tensioning device is deviated from that axis, which may occur while inserting an interference screw, strand tension may alter. This may have an adverse impact on the biomechanical properties of the graft, which in turn may affect the surgical outcome.

This study aimed to quantify the effects, on individual strand tension and stress, on tensioning device mal-positioning. The null hypotheses were as follows:

1. Individual strand tensions, during looped four-strand tendon graft ACL reconstruction, are equal when using a tensioning device in line with the longitudinal axis of the tibial tunnel.

2. Angulation of the tensioning device, with respect to the long axis of the tibial tunnel, will result in equal strand tension.

3. Locking the tensioning device at extreme angles will result in equal strand tension.

## Methods

Simulation of ACL reconstruction with a looped four-strand tendon graft was performed using cadaveric bovine tibiae and sheep superficial digital flexor (SDF) tendons harvested from skeletally mature individuals. The utilization of animal-derived tissues was approved by the Institutional Animal Welfare Committee.

To obtain a study power of 0.8 with an alpha of 0.05, the required sample size was determined to be n = 20. To this end 20 cadaveric reconstructions were performed and tested.

**Figure 1:**
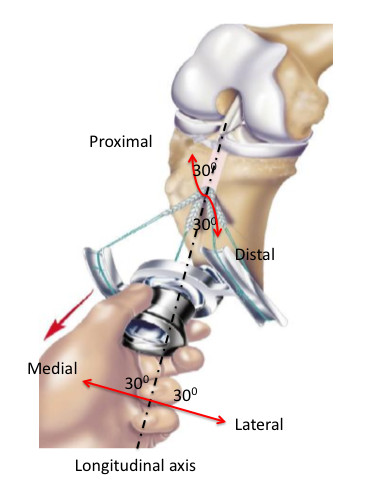
**Schematic showing approximate position of the strand bundle when undergoing tensioning in the various planes**. This figure does not reflect tensioning device 'locking state'.

The ACL Tie Tensioner (Mitek, Johnson and Johnson, USA) was evaluated for its ability to apply reproducible individual strand tension when positioned as might occur in clinical practice (Figure 1).

Retrieved tendon strands were whipstitched using No. 1 braided polyester suture (Ethibond, Ethicon, Inc., USA); Suture loops were attached to hooks connected to each load cell. The diameter of the graft composite was measured by passing it through an incremental sizing block to achieve a bundled strand diameter of 8.00 mm. The femoral aspect of the graft was stabilized at the level of the tibial plateau using a circular rod passed through the centre of the tendon loops and which rested on the tibial plateau.

**Figure 2:**
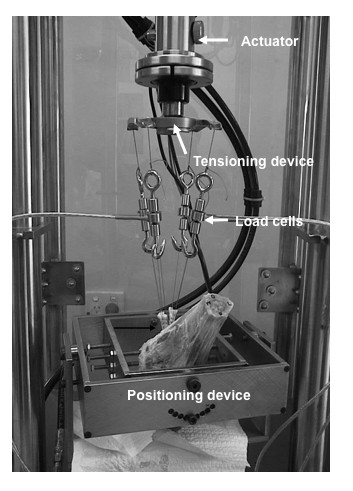
**The component arrangement for testing of reconstructions**: The tensioning device is positioned in series with the reconstruction, four load cells and the load-train of the Instron as depicted.

Biomechanical tests were performed with an Instron materials testing system (Instron Pty Ltd, High Wycombe, UK). Once placed in the testing system, with the tibial tunnel at zero degrees (vertical), each tendon suture loop was attached to a 25 kg (223 N) load cell (AL Design Inc, Buffalo, New York, USA model ALD.75 DIA UTC MINI-50 lb). All four load cells were then attached to the tensioning device such that each arm supported two tendons and their accompanying load cells (Figure 2). Load cells were then balanced before applying tension to the tendon strands. These were loaded to 150 N in tension for 10 sinusoidal cycles at 0.1 Hz., allowing the tendons to reach a steady state of hysteresis and reduce the effects of creep and stress relaxation found in viscoelastic tissue. Once completed the Instron was kept in load control to maintain a tension of 150 N on the tendons.

The tibia was then moved to place the tunnel at the positions described below. At each position, the Instron load was allowed to return to 150 N. The position was maintained for five seconds before moving to the next position. This allowed time for the tendons to undergo creep recovery from their prior location and which also served as a reference point for the next sequence of tibial tunnel angulations.

**Figure 3:**
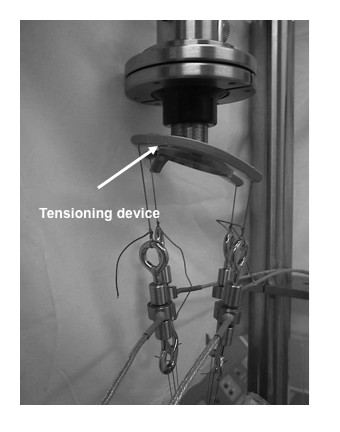
**Tensioning device locking state**: The arms of the tensioning device are shown in the locked clockwise position with the central ring firmly pressed against the tensioning tube.

The tensioning device was first evaluated in the unlocked position (longitudinal alignment with axis of tibial tunnel) then locked clock wise (CW) followed by counter-clockwise (C-CW) locking (Figure 3). The tests were repeated at each of the seven predetermined positions (tensioning device angle) for each of the locking states.

The load in each tendon strand and actuator displacement, was recorded for subsequent data analysis. Statistical analysis was performed with SPSS (SPSS Inc., Illinois, USA). Repeated measures analysis of variance (ANOVA) was used to evaluate the data. The independent variables, tensioning device state (unlocked, locked clockwise, and locked counterclockwise), tensioning device position (7 positions, 01, D30, P30, 02, M30, L30, 03) and tendon position (4 positions; bottom lateral [BL], top medial [TM], top lateral [TL] and bottom medial [BM]) were considered as within-subject factors. The dependant variable was the tension in each strand. For all statistical comparisons, a probability level of p < 0.05 was considered significant.

## Results

**Figure 4:**
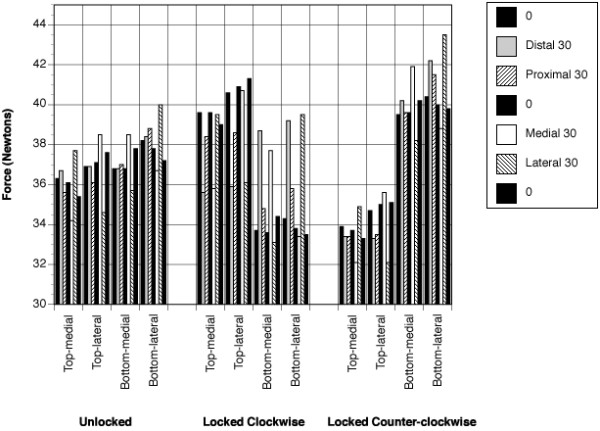
**Strand tension**: Graphical representation of individual strand tension (Newtons - N) in response to tensioning device and tendon position. Standard deviations are not assigned to the figure to reduce its complexity.

Mean strand tensions for each test are displayed in Table 1 and presented graphically in Figure 4. These provide a synopsis of the strand bundle response to each of the positions adopted and also reflect the 'locking state' of the tensioning device.

**Table 1:** The data displayed represents the mean strand tension (Newtons) ± standard deviation compiled from testing each reconstruction (n = 20)

Unlocked	01	D30	P30	02	M30	L30	03
Top-medial	36.3 ± 1.4	36.7 ± 2.2	35.6 ± 1.6	36.1 ± 1.9	34.2 ± 1.8	37.7 ± 2.0	35.4 ± 1.7

Top-lateral	36.9 ± 1.6	36.9 ± 2.6	36.1 ± 2.5	37.1 ± 1.8	38.5 ± 2.7	34.6 ± 2.3	37.6 ± 1.7

Bottom-medial	36.8 ± 3.1	36.8 ± 2.7	35.6 ± 1.6	36.8 ± 2.8	38.5 ± 2.9	35.6 ± 2.4	37.8 ± 2.3

Bottom-lateral	38.1 ± 3.0	38.3 ± 2.8	38.8 ± 2.9	37.8 ± 2.9	36.7 ± 2.9	40.0 ± 2.3	37.2 ± 2.6

**Locked-clockwise**	**01**	**D30**	**P30**	**02**	**M30**	**L30**	**03**

Top-medial	39.6 ± 1.4	35.6 ± 3.2	38.4 ± 2.1	39.6 ± 1.6	35.8 ± 2.4	39.5 ± 2.2	39.0 ± 1.5

Top-lateral	40.6 ± 2.2	35.9 ± 3.3	38.6 ± 3.7	40.9 ± 1.3	40.7 ± 2.9	36.1 ± 3.3	41.3 ± 1.3

Bottom-medial	33.7 ± 3.0	38.7 ± 5.2	34.8 ± 3.2	33.6 ± 2.5	37.7 ± 3.1	33.1 ± 2.2	34.4 ± 2.3

Bottom-lateral	34.3 ± 3.1	39.2 ± 3.0	35.8 ± 3.5	33.8 ± 2.7	33.4 ± 3.0	39.5 ± 3.4	33.5 ± 2.8

**Locked-counterclockwise**	**01**	**D30**	**P30**	**02**	**M30**	**L30**	**03**

Top-medial	33.9 ± 1.9	33.4 ± 2.2	33.4 ± 2.2	33.7 ± 1.8	32.1 ± 2.5	34.9 ± 2.1	33.3 ± 1.6

Top-lateral	34.7 ± 1.9	33.3 ± 2.4	33.5 ± 3.6	35.0 ± 1.4	35.6 ± 2.6	32.1 ± 2.8	35.1 ± 1.5

Bottom-medial	39.5 ± 2.9	40.2 ± 3.3	39.6 ± 2.3	39.6 ± 2.7	41.9 ± 2.8	38.2 ± 2.3	40.2 ± 2.3

Bottom-lateral	40.4 ± 2.6	42.2 ± 2.9	41.5 ± 3.7	40.0 ± 2.1	38.8 ± 3.6	43.5 ± 2.1	39.8 ± 2.5

Testing was performed with the tensioning device held in three locking states (unlocked, locked-clockwise and locked-counterclockwise. Individual strand response to loading (top-medial, top-lateral, bottom medial and bottom lateral) are recorded at each position (01, 02, 03: neutral; D30, P30: distal or proximal excursion; M30, L30: lateral or medial excursion).

When the tensioning device is utilized in the unlocked position (aligned with the longitudinal axis of the tunnel), the angle at which the tensioning device is held produces a significant effect (p < 0.0001) on the outcome measures. Conversely, tendon position does not produce a significant effect (p = 0.051). The interaction between tensioning device angle and tendon position is significant (p < 0.001) with BL significantly greater than TM at all angles (p < 0.025).

The results of tensioning device locking produced significant main effects with tensioning device angle (p < 0.001), locking state ((p < 0.046) and tendon position (p < 0.016) all producing significant effects on the resultant strand tension. The interaction between tensioning device angle and tendon position was significant (p < 0.001) as was locking state and tendon position ((p < 0.001) with the interaction between locking state, tensioning device angle and tendon position also producing significant effects (p < 0.001)

## Discussion

ACL reconstruction with the looped four-strand tendon graft has gained popularity. Although clinical outcomes [4,15,17,18] are similar to BPTB grafting there appear to be fewer complications with optimal fixation techniques now available [13,14]. The distribution of tension in all strands of the graft, an integral factor in its success, is gaining widespread attention [15,18]. Due to the composite nature of the graft, it appears essential to apply equal tension to all the strands during tibial fixation [8,15]. This, it is suggested, will provide optimal strength and stiffness to the graft leading to a better surgical outcome [5,6,22]. It is further proposed that any disparity in the tension between strands may lead to disproportionate tensile loading and which, may ultimately lead to early rupture of the strands, weakening the entire reconstruction.

Brown et al [23] evaluated the manual application of tension to grafts followed by fixation with 4.5 mm cortical screws in combination with plastic, spiked washers. In order to produce equal tension in all four strands, suture loops were created from the graft ends; no data was presented to confirm equality in strand tension. Hammer et al [10 ], produced equal tension in strands by applying known weights. This study showed that when strands were clamped, they exhibited better tensile properties. The mean maximum load obtained for four strand grafts was 2831 ± 538 N when the tension had been applied manually and 4590 ± 674 N when it had been applied with a weight. However, tension in individual strands was not documented.

The objective in performing this cadaveric study was to quantify the level of tension applied to all strands of a looped four-strand tendon graft before tibial fixation. This was undertaken to investigate the impact of mal-positioning of the tensioning device on the resultant strand tension. The analysis was conducted at three neutral positions (01, 02, and 03) and with the tensioning device helf in various positions (medial and lateral excursion - 30 ^0^; proximal and distal excursion - 30 ^0 ^) and locking states (Figures 1, 2, and 3).

Our first null hypothesis was shown, in part, to be correct; strand tensions were not significantly different when the tensioning device was at the 01 and 02 neutral positions. However, strand tension differed significantly at the third neutral position, 03 (Figure 4). One possible explanation was that this position (03) followed medio-lateral excursion of the tensioning device which may have indiced residual tendon deformation, altering their biomechanical behavior.

Our second null hypothesis evaluated the effect of extreme angulation of the tensioning device, when deviated to 30° from the neutral position in all four planes, in the unlocked state (Figure 4). Strand tensions were recorded at four positions; distal 30 (D30), proximal 30 (P30), medial 30 (M30) and lateral 30 (L30). Minimal variations in strand tension were observed when data was recorded with either proximal or distal excursion of the strands. A possible interpretation is that at D30 and P30 the tendons are deviated proximally and distally from their longitudinal axis, which may have reduced impact on changes to their biomechanical properties. The plane of proximal-distal rotation lies closer to the longitudinal axis of the tunnel and hence the tendons.

Conversely, strand tension showed a significant differences when the tensioning device was deviated to M30 and L30 allowing rejection of the second null hypothesis in this specific situation. A possible explanation is that at M30 and L30 there is medio-lateral excursion of the tendons away from their longitudinal axis. Such a variation, in the direction of load application, may result in significant structural deformation of the tendons, which in turn will have a tangible impact on their biomechanical behavior.

We rejected our third null hypothesis in that locking state of the tensioning device produced a significant impact on strand tension (Figure 4). The locked counter-clockwise state showed a greater significant difference to its other locked counterpart. A possible reason could be the shifting of the body of the tensioning device in relation to its arms, as occurs during locking; this may impact on the direction of tension transmission during tensioning. In the unlocked state, the body of the device is positioned centrally between the arms. This arrangement may contribute to a more uniform distribution of strand tension. However, when the device is locked, the body of the device moves in proximity to either the proximal or distal end of the arms, depending on the locking state. Hence, in the clockwise direction, with the tendons situated proximally, TM and TL may experience greater tensile force (39.6 and 40.6 N) as the arms of the device move away from them. In distally positioned tendons, BM and BL, may be subject to lesser tension (33.7 and 34.2 N) as the arms move towards them. Their alignment with the tunnel axis was altered, and they were displaced distally by the moving arms. Thus, locking the device causes significant variation in strand tension, which may influence the biomechanical behavior of the graft strands.

Although the strand tensions in the unlocked state were uniformly distributed, stresses were not equal because strand cross-sectional areas were different. Strand cross-sectional area had a significant impact on the resultant stress generated within the strand. Inequality of stresses may lead to early rupture of the smaller strands as they bear the greater tension per unit area. A possible solution may be the harvest of tendons having similar cross-sectional area. This would allow distribution of stresses more uniformly across all strands, ultimately providing a more optimal mechanical environment for the composite graft.

Their remains no agreement regarding the quantification of tissue viscoelasticity nor reliable modelling [24]; difficulty arises in the delineation of viscoelastic and pre-conditioning effects, as both are manifest by similar response features. The efficacy of anterior cruciate ligament reconstruction, using either QHT or BPTB grafts is thought to depend on the relative amounts of graft elongation or creep; hysteresis and creep effects appear highest during the first few loading cycles with more than 160 cycles required to reach a steady state, beyond which there was no further creep and hysteresis almost constant [25,26]. It appears that the effect of cyclic pre-conditioning is the progressive recruitment of fibres [23,26].

In the current study we have arbitrarily chosen to allow a 5 second period of relaxation between tests; this may lead to conjecture regarding our experimental methodology and possible impact of creep on the resultant data. It has been shown [27] that contraction duration significantly affects tendon strain at all levels of applied force. In response to these findings it is appropriate, in order to compare tendon mechanical properties, that the duration of loading be standardized as it has been in the current study.

A recent study [28], further complicates the situation with the suggestion that equal-stress tensioning may provide an alternative to equal-tension tensioning as performed in the current study; data derived suggested that equal-stress tensioning of tendon grafts resisted graft creep significantly better, raising the issue of the utilization of graft material having equal cross sectional areas.

## Conclusion

The findings of this study provide useful information for ACL reconstructive surgery, in which a looped four-strand tendon graft is utilized. It appears, that the optimal position to induce and maintain uniform strand tension, with a tensioning device, is along the longitudinal axis of the tibial tunnel. Any deviation from this axis, more so in the medial and lateral planes, appears to result in a significant variation in strand tension. Similarly, superior strand tension was obtained by maintaining the tensioning device in an unlocked state.

This study is a simulation of the human surgical procedure for graft tensioning. The reconstructions performed in this study, using animal-tissue, do not therefore provide a completely analogous system for comparison. However, it does appear that surgeons should consider closer attention to optimal alignment of tensioning devices in use; if this is done, a more uniform distribution of forces may be generated in the four loop components of the QHT reconstruction providing augmented mechanical characteristics of the reconstruction and, by implication, possibly improve graft longevity and effectiveness.

## Competing interests

The authors declare that they have no competing interests.

## Authors' contributions

Authors contributed variably to the concept, design and performance of this study. All authors have read and approved the final manuscript.
